# Obesity is an independent risk factor for pre-transplant portal vein thrombosis in liver recipients

**DOI:** 10.1186/1471-230X-12-114

**Published:** 2012-08-21

**Authors:** Rosa Ayala, Silvia Grande, Rosalía Bustelos, Carmen Ribera, Alvaro García-Sesma, Carlos Jimenez, Enrique Moreno, Joaquín Martínez-López

**Affiliations:** 1 Hematology Department, 12 De Octubre University Hospital, Avenida Córdoba s/n, 28041, Madrid, Spain; 2 General Surgery Alimentary Tract and Abdominal Organ Transplantation Department, 12 De Octubre University Hospital, Madrid, Spain; 3 Hematology Department, Sureste Hospital (Arganda), Madrid, Spain; 4 Complutense De Madrid University, Madrid, Spain

**Keywords:** Thrombophilia, Portal vein thrombosis, Liver transplant recipient

## Abstract

**Background:**

Portal vein thrombosis is a frequent complication in end-stage cirrhosis with a considerable peri-operative risk for liver transplant candidates. We aimed to characterize the pre-transplant portal vein thrombosis in a cohort of liver transplant recipients, and to identify independent risk factors for this complication.

**Methods:**

380 consecutive primary orthotopic liver transplants were performed in the Digestive Surgery Department of “12 de Octubre” Hospital (Madrid, Spain), between January 2001 and December 2006. The main risk factors considered were smoking, obesity, metabolic disorders, previous immobility, surgery or trauma, nephrotic syndrome, associated tumor, inflammatory disease, neoplasm myeloprolipherative. Furthermore we have reported genetic thrombophilia results for 271 recipients.

**Results:**

Sixty-two (16.3%) patients developed pre-transplant portal vein thrombosis and its presence had no impact in the overall survival of liver recipients. Obesity was the only independent risk factor for pre-transplant portal vein thrombosis.

**Conclusion:**

We recommend close control of cardiovascular factors in patients with liver cirrhosis in order to avoid associated thrombosis.

## Background

Portal vein thrombosis (PVT) is a well recognized complication in patients with end-stage cirrhosis, and its incidence ranges from 2 to 26% in different series [1]. PVT has various causes. The cause may be local, such as cirrhosis, primary or metastatic liver cancer, pylephlebitis, vascular abnormalities, and pancreatitis. Alternatively PVT may result from a thrombophilic condition such as myeloproliferative disease, Protein C (PC), Protein S (PS) and Antithrombin (AT) deficiency, or Factor V Leiden and Factor IIG20210A carriers, or a combination of a primary thrombophilia milieu that triggers the formation of the thrombus in the portal circulation [2].

PVT continues to be associated with a considerable peri-operative risk for liver transplant candidates [3,4], and is associated with increased operative time, transfusion requirements, re-interventions, and lower survival rate relative to PVT extension [5]. Therefore, PVT has been seen as an obstacle to orthotopic liver transplantation (OLT).

We aimed to identify possible parameters that could lead to PVT in a cohort of liver transplant recipients. The influence of this thrombosis in the survival of liver recipient was also evaluated.

This work is related to our previous publication [6] where we analyzed the post-transplant thrombotic events in a cohort of liver transplant recipients to identify possible parameters that could lead to HAT and could be responsible for graft loss due to thrombosis in a extended thrombophilia study. Our results showed that high fibrinogen and decreased protein C levels were parameters associated with allograft thrombosis. In the present study we analyzed the factors related to portal vein thrombosis in the pre-transplant period and their prognostic implications.

## Materials and methods

### Patient population

Between January 2001 and December 2006, 380 primary orthotopic liver transplants (OLT) were performed in the Digestive Surgery Department of the 12 de Octubre Hospital (Madrid, Spain). Wherever possible all patients are lab tested for genetic hypercoagulation study irrespective of any presence of thrombophilia. We have reported genetic thrombophilia and JAK2 mutations results for 271 primary OLT recipients. The study was approved by our Institutional Review Board and all patients gave their consent for blood samples to be further processed. Data on thrombophilia risk factors were collected by personal interview, or by reviewing the medical history.

### Recipients

The study population comprised 254 males and 126 females. The cause of liver transplantation was: 18 HBV (hepatitis B virus), 73 HCV (hepatitis C virus), 52 HBV and HCV, 62 Ethanol, 8 Autoimmune, 8 Toxicity, 29 Tumor, 63 HBV or HCV and tumor, 2 Vascular, 26 Alpha 1 antitrypsin deficits, 24 Biliary atresia and 15 cryptogenetic.

## Methods

### Genetic thrombophilia study

The extracted genomic DNA was processed to detect Factor V Leiden, prothrombin G20210A and methylenetetrahydrofolate (MTHFR) C667T mutations, using real-time PCR (polymerase chain reaction) with hybridization probes in a light-cycler (Roche Diagnostics, Mannheim, Germany).

### JAK2 mutations study

JAK2 V617F ASO quantitative PCR (ASO qPCR) was performed with the ABI PRISM 7900 (Applied Biosystems, Palo Alto, CA, USA), and a forward ASO primer spanning of the JAK2 V617F mutation region. A reverse primer and MGB TaqMan probe were also employed [7].

#### Follow-up

The follow-up period for recipients began on the date of liver transplantation, and ended on the date of death, date of re-transplant, or the end of study (March 1, 2007). Follow-up periods ranged from 15 d to 6.9 y (median: 20.23 months).

#### Statistical analyses

The Pearson’s chi-square (*χ*2) statistic and the Student’s *t* test were used to test for differences in the distribution of dichotomous variables, and for differences in the mean values of continuous distributions. Forward stepwise logistic regression was used to identify independent risk factors for PVT. The following variables collected at diagnosis were included in the database: gender (male/female), age (both as a continuous variable, and grouping patients over and under 16 years of age), the existence of clinical thrombophilia risk factors and the original liver disease.

Overall survival (OS) was calculated from the day of the liver transplantation to death. Kaplan-Meier life tables were constructed for survival data and were compared by means of the log-rank test. A census of the surviving patients was taken on March 1, 2007. Results with a *P* value less than 0.05 were considered significant.

## Results

### Thrombophilia in the study population

*Genetic study of thrombophilia.-* The prevalence of the heterozygote Factor V Leiden mutation was 7 of 271 (2.6%), the heterozygote G20210A prothrombin mutation was 13 (4.8%), and the homozygote C677T MTHFR mutation was 39 (14.9%). No correlation was observed between pre-transplant PVT and the genetic thrombophilia study (Table 1).

**Table 1:** Genetic study in association with pre-transplant PVT in liver recipients

	**With Pre-transplant PVT (50 cases)**	**Without Pre-transplant PVT(221 cases)**	**Significance**
**RECIPIENT STUDY**			
**Factor V Leiden mutation(Nº)(heterozygous) in recipient**	1/49	6/215	p = 0.774
**Prothrombin 20210A mutation(Nº)(heterozygous) in recipient**	1/49	12/209	p = 0.305
**C677T MTHFR mutation (Nº) (wild type/heterozygous/homozygous) in recipient**	18/23/7	87/93/33	p = 0.865

The study was conducted in 271 liver recipients. The MTHFR C677T genotype was not tested in ten cases. *PVT*, portal vein thrombosis; MTHFR, 5,10-methylene-tetrahydrofolate reductase.

*JAK2 mutation in liver recipients.-* JAK2 V617F was only detected in four of the 271 primary OLT recipients included in this study, details as follows: one patient diagnosed previously with PV, who presented Budd-Chiari syndrome (BCS); an other diagnosed with Budd-Chiari with features of NMP; and two others with neither thrombotic complications nor NMP features (Table 2). Two JAK2 V617F-positive recipients without thrombotic complications did not develop overt MPN after a median follow-up of nearly 4 years.

**Table 2:** Clinical features of JAK2 V617F positive liver transplant recipients

**Diagnosis**	**JAK2 V617F Allele burden (%)**	**Age**	**Gender**	**Hemoglobin (g/dL)**	**Hematocrit (%)**	**MCV (ƒL)**	**WBC count/L**	**Platelet count/L**	**Follow-up (months)**
**Alcohol cirrhosis and HCV cirrhosis**	6.71	62	M	13.9	39.4	93.8	5.50 x 10^9^	250 x 10^9^	48
**HCV cirrhosis**	4.25	72	F	10.8	35.9	84.9	5.4 x 10^9^	213 x 10^9^	60
**Budd-Chiari syndrome**	5.65	42	M	18.7	59.1	93.4	7.1 x 10^9^	234 x 10^9^	60
**Budd-Chiari syndrome and PV**	8.28	41	F	10.3	23.7	86.7	23.7x 10^9^	282 x 10^9^	38

*HCV*, hepatitis C virus; *HBV*, hepatitis B virus; *PVT*, portal vein thrombosis; M, male; F, female; *MCV*, mean corpuscular volume; *WBC*, white blood cells; *PV*, polycythemia vera.

### Pre-transplant PVT in liver recipients and clinical thrombophilia risk factors

Pre-transplant PVT was detected in 62 of 380 primary OLT recipients (16.3%). In this study population, the primary, clinical, pro-thrombotic risk factor for liver thrombosis is the existence of terminal cirrhosis. In our study, other risk factors associated with thromboses (such as the existence of diabetes or lipid alteration and obesity) were most frequent in the group with pre-transplant PVT (24 cases of diabetes or lipid alteration out of 49 with pre-transplant PVT, versus 68 of 203 without pre-transplant PVT, *P =* 0.043; and 5 cases of obesity out of 42 with pre-transplant PVT, versus 4 of 182 without pre-transplant thrombotic event, *P =* 0.004) (see Table 3). Neither the presence of tumour (*P* = 0.951), nor smoking (*P* = 0.169), were associated with pre-transplant PVT. Nevertheless child recipient was less frequent in the group with pre-transplant PVT (2 cases of 62 recipients with PVT, versus 48 of 318 recipients without PVT, p = 0.011) (Table 3).

**Table 3:** Clinical thrombophilic risk factors in association with pre-transplant PVT cases

	**With pre-transplant PVT (n = 62)**	**Without pre-transplant PVT (n = 318)**	**Significance**
Child/adult recipient	2/60	48/270	**p = 0.011**
Male/female recipient	44/18	210/108	p = 0.451
Associated tumor (Yes/No)	15/32	63/133	p = 0.951
Diabetes mellitus and/or high lipid (Yes/No)	24/25	68/135	**p = 0.043**
Inflammatory disease (Yes/No)	0/39	0/183	p = N
Smoking (Yes/No)	20/26	111/92	p = 0.169
Inmobility (Yes/No)	3/36	9/166	p = 0.531
Surgery Intervention (Yes/No)	24/23	104/103	p = 0.919
Obesity (Yes/No)	5/37	4/178	**p = 0.004**
Nephrotic syndrome (Yes/No)	0/39	14/163	p = 0.069
Myeloproliferative syndrome (Yes/No)	2/39	0/185	**p = 0.003**
Trauma (Yes/No)	3/37	10/171	p = 0.631
Cause of liver transplantation			p = 0.135
BHV	2	16	
CHV	12	61	
Ethanol	14	48	
Autoimmune	3	5	
Toxicity	1	7	
Tumor	7	22	
Cryptogenetic	1	14	
Vascular	2	0	
Infrequent (deficit alpha1 antitrypsin)	3	23	
Biliary atresia	2	22	
BHV and CHV	10	42	
BHV and CHV and Tumor	5	58	

*HCV*, hepatitis C virus; *HBV*, hepatitis B virus; *PVT*, portal vein thrombosis. Data on height was available in 224 of 380 recipients. The body mass index (BMI) was calculated as weight in kilograms/height in meters squared. Obesity was considered when BMI > 30 kg/m2. Missing Values: associated tumor (138); DM and/or High lipid (128); Inflammatory disease (158); Smoking (131); Inmobility (166); Surgery Intervention (126); Nephrotic Syndrome (164); Myeloproliferative Syndrome (159); Trauma (159).

### Multivariate study

In the multivariate study, the only variable associated with pre-transplant PVT was obesity (no/yes) (HR 13.2, *P* < 0.02) (Table 4).

**Table 4:** Logistic Regression analysis of factors associated with pre-transplant PVT

**Variable**	**p**	**Hazard Ratio (CI)**
AGE (CONTINUOUS VARIABLE)	0.119	
OBESITY (YES/NO)	**0.016**	**13.161 (1.324-130)**
ASSOCIATED TUMOR (YES/NO)	0.756	
LUPUS (YES/NO)	0.766	
SMOKING (YES/NO)	0.125	
DIABETES MELLITUS AND/OR HIGH LIPID (YES/NO)	0.115	
CAUSE OF LIVER TRANSPLANTATION	0.184	
FACTOR V LEIDEN	0.728	
PROTHROMBIN 20210A MUTATION	0.237	
MTHFR C677T MUTATION	0.377	

Obesity (no/yes) (HR 14.7, P < 0.02) was associated with pre-transplant PVT.

### Survival analysis

**Figure 1:**
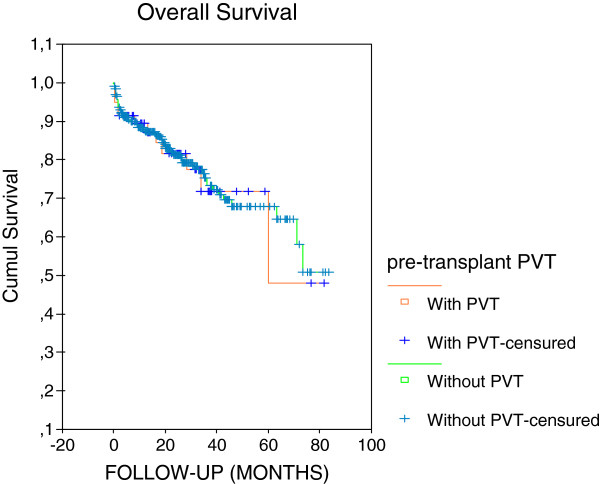
**Recipient Overall Survival.** Kaplan-Meier analyses of OS with respect to the presence of pre-transplant PVT. There was no association of pre-transplant PVT with recipient OS (P = 0.989). OS was not different between liver recipients with pre-transplant PVT (n = 59 evaluated cases; 79.18%) and without pre-transplant PVT (n = 317; 79.66%). 4 cases were not evaluated for OS. Cumul Survival (Cumulative Survival); pre-transplant PVT (pre-transplant portal vein thrombosis).

Kaplan Meier tests were performed (Figure 1) and the results indicated that pre-transplant PVT was not associated with a poor outcome.

## Discussion

During a liver transplantation, pre-transplant PVT, encountered in approximately 15% of candidates, increases surgical difficulties and post-operative PV re-thromboses [8]. However, there is no extensive thrombophilia study in a large cohort of liver recipients. In our study, all cases are characterized by the presence of local thrombophilia risk factors: cirrhosis and other factors such as primary cancer, vascular abnormalities. We found obesity as the only clinical factor associated with pre-transplant PVT. Previously obesity has been considered to be a risk factor for the venous thromboembolism as well as arterial thrombosis [9-15], and was also previously reported as an independent risk factor for clinical decompensation in patients with cirrhosis [16] but it had not been previously associated with PVT. Plausible mechanisms to explain the relation between obesity and venous thrombosis include the existence of a proinflammatory, prothrombotic, and hypofibrinolitic milieu in the obese patients [12,13].

The importance of genetic thrombophilia risk factors in PVT has been investigated in several studies [17-20] but all of them have excluded cases with local risk factors. The incidence of Factor V Leiden, prothrombin mutation, decreased PC, decreased PS, and decreased AT has been recorded between 3-30%, 3-22%, 0-26%, 2-43%, and 1-26%, respectively. In our study the incidence of Factor V Leiden and prothrombin mutation, was 2.4% and 4.5%, respectively. The incidence of Factor V Leiden and prothrombin mutations coincide with that seen in the general population and this fact could prove that genetic thrombophilia has no role in pre-transplant PVT in patients with cirrhosis.

JAK2 V617F testing represented an important advance in the diagnostic workup for the recognition of atypical myeloproliferative disease in BCS and PVT patients [21]. Our results do not support the routine screening of JAK2 V617F mutation in patients with splanchnic thrombosis in terminal cirrhosis or malignancy, except when patients have Budd-Chiari syndrome. Our results do not contradict Plessier et al where patients with cirrhosis or malignacy were specifically excluded [22]. Unlike previous studies [3,23,24],the presence of pre-transplant PVT did not influence the overall survival of recipients. Other authors also reported no significant differences between both groups for 1- to 10-year patient survival [25-28].

## Conclusions

To the best of our knowledge, this is the first extensive genetic thrombophilia study in a large cohort of liver recipients. Obesity and diabetes mellitus and/or hyperlipidaemia were associated with pre-transplant PVT, but obesity was the only independent risk factor for pre-transplant PVT. We stress the importance of controlling the cardiovascular factors in patients with liver cirrhosis.

## Competing interests

The authors have no conflicts of interest to declare.

## Authors’ contributions

Contribution: RA and JML designed research; SG and RB carried out the molecular genetic studies; RA, JML, AGS, CJ, EM, and CR analyzed and interpreted data; RA designed, performed, and wrote the manuscript; JML supervised the research and critically revised the manuscript. All authors read and approved the final manuscript.

## Pre-publication history

The pre-publication history for this paper can be accessed here:

http://www.biomedcentral.com/1471-230X/12/114/prepub
